# Preparation and characterization of carex meyeriana Kunthcellulose nanofibers by electrospinning

**DOI:** 10.1038/s41598-022-25835-6

**Published:** 2022-12-23

**Authors:** Ying Sun, Yang Yu, Duanxin Li, Weishuai Kong, Feng Yang

**Affiliations:** 1 College of Light Industry and Textile, Qiqihar University, Qiqihar, 161000 Heilongjiang China; 2 Engineering Research Center for Hemp and Product in Cold Region of Ministry of Education, Qiqihar University, Qiqihar, 161006 China

**Keywords:** Materials science, Nanoscience and technology

## Abstract

The cellulose of carex meyeriana kunth (CMKC) was used as raw material, and the spinning solution was prepared by combining with polyacrylonitrile (PAN). The nano-cellulose fiber of carex meyeriana kunth (CMKN) was prepared by electrospinning. Used to remove methylene blue dye (MB) in aqueous solution. In the electrospinning experiment, the addition of CMKC was in the range of 5% ~ 25%, the feed rate of spinning parameters was set in the range of 0.2 ~ 1.0 mL/h, the distance from the needle tip to the collecting plate was in the range of 10 ~ 25 cm, and the voltage was changed in the range of 15 ~ 25 kV. The obtained CMKN was characterized by scanning electron microscope, X-ray diffraction (XRD) and Fourier transform infrared spectroscopy. The MB removal rate was evaluated in the dye removal experiment, and the effects of CMKN on MB removal rate under the factors of CMKC dosage, temperature, shock time and MB initial concentration were discussed. The optimum process conditions were determined by response surface methodology. The results show that the prepared fibers are superfine fibers with nanometer diameter, and the spun nanofibers have smooth surface, high overall orientation and strong uniformity. The adsorption kinetics of prepared CMKN accords with quasi-second order model, and the adsorption isotherm accords with Langmuir model. The maximum dye removal rate of CMKN is 63.24%.

## Introduction

Nowadays, with the aggravation of ecological environment pollution and over-exploitation of fossil energy, the earth is facing a severe energy shortage problem. It is necessary to find other resources to relieve the pressure of over-exploitation of fossil energy and adapt to the needs of human life. With the research in recent years, it has been found that natural cellulose in nature has many excellent characteristics, such as reproducibility, biodegradability, biocompatibility and thermal stability, etc., and it is a kind of green, environmentally friendly and sustainable natural resource^1–3^. Among them, Carex meyeriana Kunth is a kind of cellulose resource which has not been fully and effectively developed in nature.

Carex meyeriana Kunth belongs to carex of monocotyledonous carex family. Its components are mainly composed of cellulose and part of hemicellulose, lignin and pectin^4^. And Carex meyeriana Kunth’s growth cycle is relatively short, basically one year. Carex meyeriana Kunth is widely distributed in nature. Most of them grow in swamps and wetlands where the environment is humid. It can be found in northern China, Russia, Mongolia, North Korea and other countries in the Far East^5^. This also provides a strong guarantee for its ability to supply sufficient cellulose raw materials in production research. In addition, carex meyeriana Kunth has good antibacterial, moisture absorption, ventilation, cold resistance, warmth retention and ultraviolet resistance. It is a rare new environment-friendly material^6,7^.

Because of the outstanding advantages of cellulose, the research on nanofibers from cellulose has aroused great interest^8–11^. When cellulose is combined with nano-materials to produce nano-cellulose, the original advantages of cellulose will be increased by the characteristics of nano-materials such as large specific surface area, strong mechanical properties and strong adsorption^12–14^. In recent years, there are many researches about the preparation of nano-cellulose, among which electrospinning is one of the methods.

The principle of preparing nano-cellulose by electrostatic spinning technology is that according to the characteristic that cellulose is insoluble in water but only soluble in organic solvents, appropriate organic solvents are selected to dissolve cellulose macromolecules, such as LiCl/DMAc^15^, ionic liquids^16^ and deep eutectic (DES)^17^ and other solvent systems, so as to destroy the hydrogen bond network between cellulose molecules and achieve the purpose of dissolving cellulose, and then spinning solution is prepared. Then, an externally applied electric field is used to force the injector nozzle to spray a continuous cellulose polymer solution to form a jet. When the solution jet solidifies and randomly deposits on the collector surface, nano-sized electrospun fibers will be generated. During spinning, many factors will affect electrospun fibers. For example, spinning parameters (voltage, liquid pushing speed, receiving distance and receiver type), environmental factors (temperature and humidity) and solution parameters (solution concentration, viscosity, surface tension and conductivity) all play a vital role in the formation and morphology of electrospun fibers^18–20^.

Nowadays, nano-cellulose is widely used in the fields of membrane technology, reinforcing materials, catalytic materials, adsorption and filtration, biomedicine and national defense security^21–23^. It is considered as the most important potential candidate material to replace petroleum-based polymers. In recent years, the preparation of nanofiber membrane by electrospinning technology for adsorption of printing and dyeing wastewater is also a research hotspot. Due to the increasing demand for dyes in the printing and dyeing industry, the amount of dye wastewater discharged in the production process has greatly increased. Moreover, the printing and dyeing wastewater has many problems, such as high chromaticity, complex components, poor biodegradability, and many toxic and harmful substances, all of which bring huge hidden dangers to the environment^24^.

Therefore, many researchers have studied the treatment of dye wastewater, and membrane separation technology is one of the most effective methods to remove pollutants from water. Separation can effectively remove pollutants by virtue of the properties of nanofiber membrane, such as porosity, surface charge and hydrophilic/hydrophobic properties. Duy-Nam: Polyacrylonitrile (PAN), zinc oxide and hinokitiol (HT) were prepared into composite nanofibers by electrospinning, which were used for sterilization and dye removal^25^. Abdul Sameeu prepared polyacrylonitrile-based activated carbon nanofibers by electrospinning and heat treatment, and adsorbed methylene blue dye in aqueous solution^26^. The insoluble β-cyclodextrin/glutaraldehyde crosslinked polyvinylpyrrolidone nanofiber membrane was prepared by Ning electrospinning method, and the adsorption of methyl orange (MO) dye was tested^27^.

**Figure 1:**
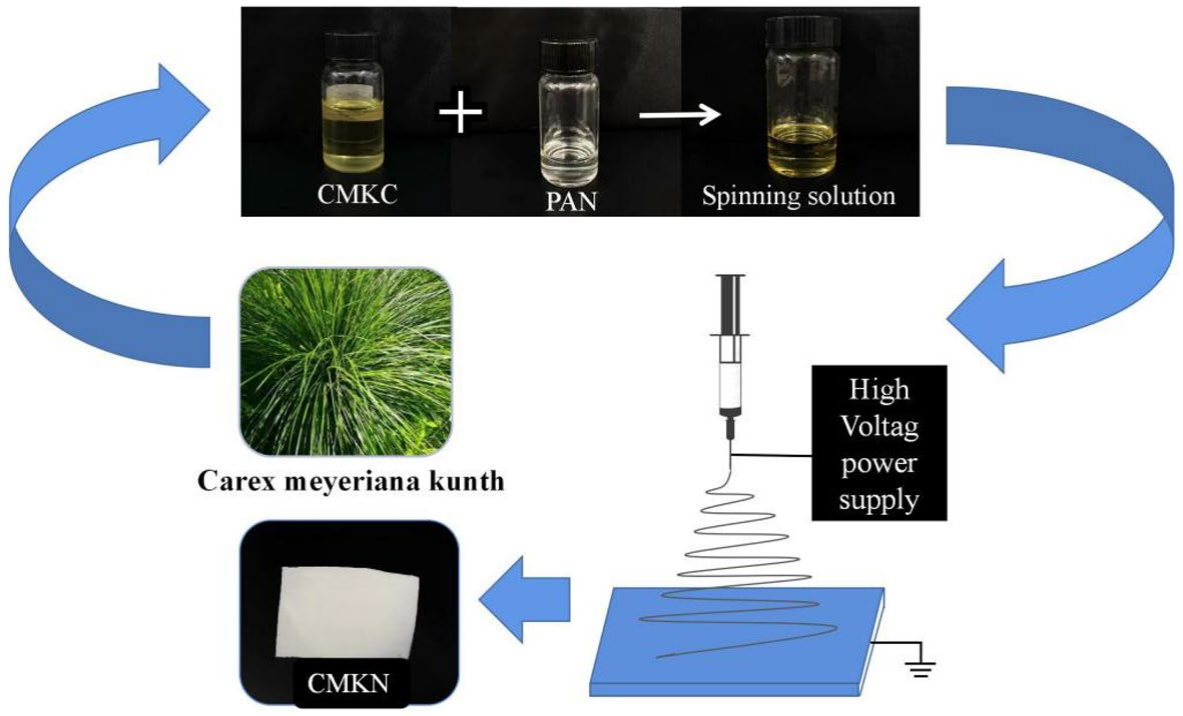
Route of electrospinning experiment.

In this experiment, carex meyeriana kunth nano-cellulose /PAN composite film (CMKN) was prepared by electrospinning technology. The experimental process is shown in Fig. 1. The effects of CMKC concentration, liquid pushing speed, voltage and receiving distance on the film-forming effect of electrospinning CMKN were explored. The methylene blue dye was used to simulate printing and dyeing wastewater, and the removal effect of CMKN on methylene blue was studied.

## Experimental

### Materials

Carex meyeriana kunth (purchased from Jiamusi City, Heilongjiang Province, China). The carex meyeriana kunth’s cellulose (CMKC) used in this study was self-made in the laboratory. The other reagents used in this experiment are as follows: N, N-dimethyl acetamide (DMAc, ≥ 99.0%, SCR) and polyacrylonitrile (PAN, with an average molecular weight of 150,000 g/mol); methylene blue (MB, ≥ 98.5%,Tianjin Zhiyuan Chemical Reagent Co., LTD). All chemicals used in this study were analytical grade reagents without any further treatment, and all solutions were prepared with distilled water.

### Preparation of cellulose nanofiber

Preparation of cellulose solution of carex meyeriana kunth.

Immerse carex meyeriana kunth in soapy water at 50 ± 2 °C for 12 h, then wash and dry. Grinding into carex meyeriana kunth powder, and sieving with 80 mesh sieves. Put the sieved powder into a fat extraction device, prepare 2:1 volume ratio of phenylethanol solution, and extract at 80 °C for 3 h. Completely washed with deionized water and dried at 50 °C to constant weight. Then, put the powder and distilled water in a beaker at a bath ratio of 1:50, and treat with sodium hydroxide-hydrogen peroxide system, taking 4 g/L of hydrogen peroxide and 8 g/L of sodium hydroxide, boiling in a water bath at 80 °C for 1 h, then completely washing the powder with deionized water, and drying at 50 °C for later use. Finally, put the dried powder and distilled water in a beaker at a bath ratio of 1:50, and adjust the PH range of the solution to 3–4 with sulfuric acid. Cook in a water bath at 90 °C for 1 h, and finally, the obtained powder is completely washed with deionized water and dried at 50 °C to constant weight. Thereby obtaining carex meyeriana kunth cellulose.

0.1 g of carex meyeriana kunth cellulose was weighed by electronic balance and dissolved in 20 ml of LiCl/DMAc solution with mass fraction of 10% in 50 ml round bottom flask. Stir with a magnetic stirring bar at 90 °C for 3 h, and then stir at room temperature for 1 h until a uniform solution is obtained.

#### Preparation of eletrospinning solutions

PAN powder was dissolved in DMAc to prepare a 15% PAN solution. The resulting solution was magnetically stirred until evenly mixed. Then, the self-made CMKC solution was mixed with 15% PAN solution in different proportions to prepare 5%, 10%, 15%, and 25% CMKC-PAN electrospinning solutions. The mixed solution was cleaned by an ultrasonic cleaning machine for a certain time to produce an evenly mixed solution.

#### Electrostatic spinning process

The evenly mixed solution was electrospun into ultrafine fibers by an electrostatic spinning equipment (YFSP-T, Yunfan electrostatic spinning machine). The parameters, such as the applied voltage, the pushing speed, and the accepting distance, can be manually adjusted via the control panel on the equipment. The applied voltage was within 15–25 kV, and the feed rate of the solution was controlled at 0.2–1 mL/h. The collector wrapped in aluminum foil was placed 10–25 cm away from the tip of the nozzle to collect the fibers. Electrospinning was performed at room temperature and 60% relative humidity. The fibers were removed from the aluminum foil and completely dried in vacuum at 65 °C.

### Response surface optimization of dye adsorption process

After carex meyeriana kunth cellulose nanofilm and methylene blue dye are dried in the drying oven, methylene blue solution of a certain concentration is configured, and then carex meyeriana kunth cellulose nanofilm is cut into small pieces of different quality and added to methylene blue solution. Use a constant temperature oscillator for a period of time to promote dye adsorption. After the adsorption is completed, the dye solution after adsorption is taken and tested in the UV–visible near-infrared spectrophotometer. The dye removal rate is calculated according to the absorbance of the residual solution.

The molecule of carex meyeriana kunth cellulose contains a large number of hydrophilic groups-hydroxyl groups. At the same time, adding H_2_O_2_ can oxidize the hydroxyl groups on the surface of CMKC to carboxyl groups, thus increasing the number of carboxyl groups on CMKN. Methylene blue is a cationic dye, which is easily attracted by negatively charged carboxyl groups on CMKN. In addition, the nanofiber membrane prepared by electrospinning method has large specific surface area and many dyes attachment points. The adsorption capacity of MB can be improved.

#### Single factor experiment

The effects of CMKC dosage, MB initial concentration, temperature and shock time on dye removal by CMKN were investigated in simulated wastewater containing methylene blue.

#### Response surface method was used to optimize the experimental design

According to the analysis of single factor experimental results, according to the design principle of Box-Behnken central combination experiment, with temperature (A), shock time (B) and MB concentration (C) as independent variables and total dye removal rate as response value, response surface analysis method with three factors and three levels was used to obtain the optimized process parameters. The design of experimental factor level is shown in Table 1.

**Table 1:** Three Factors Levels and Codes of Box-Behnken Design.

The coding level	A temperature (°C)	B shock time (min)	C MB concentration (mg/L)
− 1	25	80	30
0	30	90	40
1	35	100	50

### Ethical approval

All the methods were carried out in accordance with local, China and Qiqihar University guidelines and regulations.

## Characterizations and measurements

### SEM analysis

Scanning electron microscopy (SEM) images of the fiber surface were taken with an S-3400 (Hitachi) scanning electron microscope. The microscope was operated at 10 kV, 20 °C, and RH of 65%. Prior to SEM evaluation, the samples were coated with a thin layer of gold using a plasma sputtering apparatus.

### XRD analysis

X-ray diffraction (XRD) patterns were recorded from 2θ = 10–90° with a D/max-RB diffractometer equipped with a graphite monochromator and Cu Kα radiation at λ = 0.154 nm (45 kV, 200 mA).

### FT-IR analysis

The chemical functional groups in CMKC and CMKN were determined by infrared spectroscopy. The changes between the two were analyzed. The optical fibers were analyzed using a Spectrum One Infrared spectroscopy analyzer (PE, USA). The spectrum obtained is the result of 30 scans at 4 cm^−1^ resolution in the range of 400–4000 cm^−1^.

### Mechanical and physical test

The CMKN samples were treated in a vacuum oven (65 ± 2 °C) for 24 h and then cut into 5 mm × 3 cm strips for testing. The fiber strength and elongation at break were measured by an LY-06E fiber strength tester at 20 °C and RH of 65%. The pre-tension was 0.6 CN/dtex, and the tensile length and speed were maintained at 10 mm and 30 mm/min, respectively. The results from the five specimens were averaged^28^.

### Adsorption rate test

Using CMKN as adsorbent for static experiment, the specific operation process is: a certain mass of CMKN was added to the methylene blue solution with a certain concentration, and the methylene blue wave length was known to be about 650 nm after shaking on the digital display constant temperature oscillator for a certain time. The absorbance and concentration of the remaining dye were measured by UV spectrophotometer, and the removal rate was calculated. As shown in the following formula, C_0_: initial dye concentration (mg/L); C_1_: is the dye concentration in solution at time t (mg/L)^28^.

$$\mathrm{Dye\, removal\, rate}\,=\,\frac{{C}_{O}-{C}_{t}}{{C}_{0}} \times 100\%.$$

## Results and discussion

### SEM analysis

#### Effect of different proportions of cellulose and PAN on electrospinning

**Figure 2:**
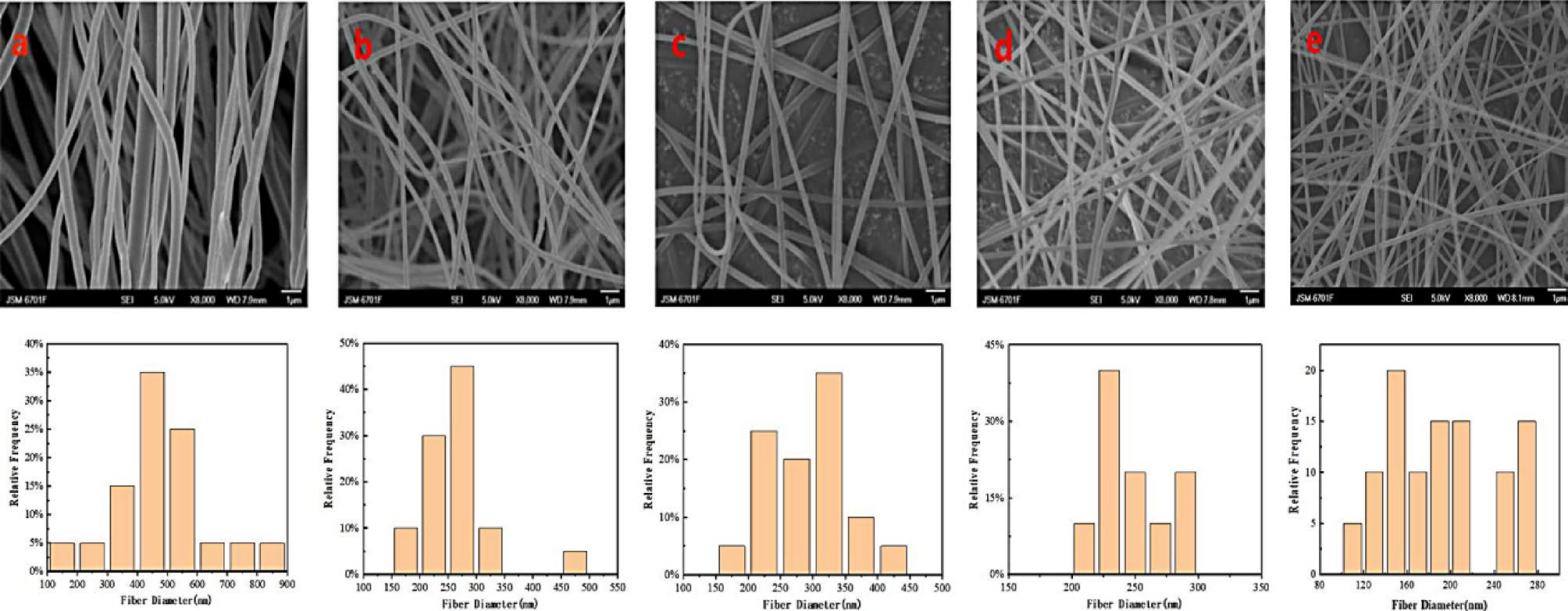
SEM images of electrospinning CMKN fibers with different cellulose ratio and fiber diameter distribution ((**a**): 5%; (**b**):10%; (**c**):15%; (**d**):25%).

In electrospinning, the spinning fluid has a very important effect on fiber formation^29^. The cellulose solution of pure carex meyeriana kunth has poor stability and low viscosity and is difficult to spin by electrostatic spinning. Therefore, a certain proportion of PAN should be used to improve the spinnability^30,31^. Figure 2 shows the SEM images of different proportions of cellulose and PAN electrospinning fibers. In this experiment, the effects of 5, 10, 15 and 25% cellulose in the cellulose and PAN mixture on the electrospinning effect were investigated. Electrospinning was performed at 18 kV, the receiving distance of 15 cm, and the propulsion speed 0.5 mL/h.

Figure 2a clearly shows that when the cellulose/PAN ratio is 5%, the diameter of the polymer fiber formed is irregular, and a large gap exists between the fiber diameters. The fiber diameter is mostly distributed between 400 and 500 nm, and the surface is rough and greatly curved. Figure 2b indicates that when the cellulose/PAN ratio is 10%, the fiber diameter distribution is mostly between 250 and 300 nm. Thus, the fiber surface is obviously smooth, but some bending remains. Some improvement can be observed compared with adding 5% cellulose. Figure 2c shows that when the cellulose/PAN ratio is 15%, the overall fiber distribution is relatively uniform under electron microscopy, and the fiber diameter is mainly concentrated in the range of 300–350 nm. The overall uniformity of fiber is high, the fiber surface is smooth and smooth, relatively continuous, without too much bending, and the spinning effect is good. Figure 2d shows that when the cellulose/PAN ratio is 25%, the fiber diameter is mainly distributed between 160 and 230 nm. Although the fiber generally becomes fine, certain beads remain, and the bending degree of the fiber increases. Thus, the spinning condition is poor. As shown in Fig. 2e. The diameters of polyacrylonitrile nanofibers are between 150 and 280 nm. The fiber has a smooth appearance and uniform diameter distribution.

When the cellulose/PAN ratio is 15%, the fiber diameter distribution is uniform, the fiber morphology difference is small, the fiber surface is flat, and the fiber fineness is good. If the concentration is extremely low, then the fiber diameter will be thick and the uniformity will be worse. Increasing the concentration to more than 25% will result in liquid beads, poor stability, and reduced spinnability. Therefore, the optimal blending ratio of 15% can be adopted for the subsequent experimental research.

#### Effect of voltage on electrospinning

Spinning voltage also has a great influence on fiber formation in electrostatic spinning^32^. If the electrostatic spinning voltage is extremely low, the effect of the electric field force in the electric field will be weakened, and the solution pushed out by the syringe will be affected by its own viscosity and surface tension resistance, so it cannot spin smoothly^33^. If the voltage of electrostatic spinning is too high and the charge load is too large, the discharge phenomenon will cause damage to the nozzle and receiver^34^. In this experiment, 15% cellulose ratio, liquid pushing speed of 0.2 mL/h and receiving distance of 20 cm were selected to study the influence of different voltages on CMKC electrostatic spinning.

**Figure 3:**
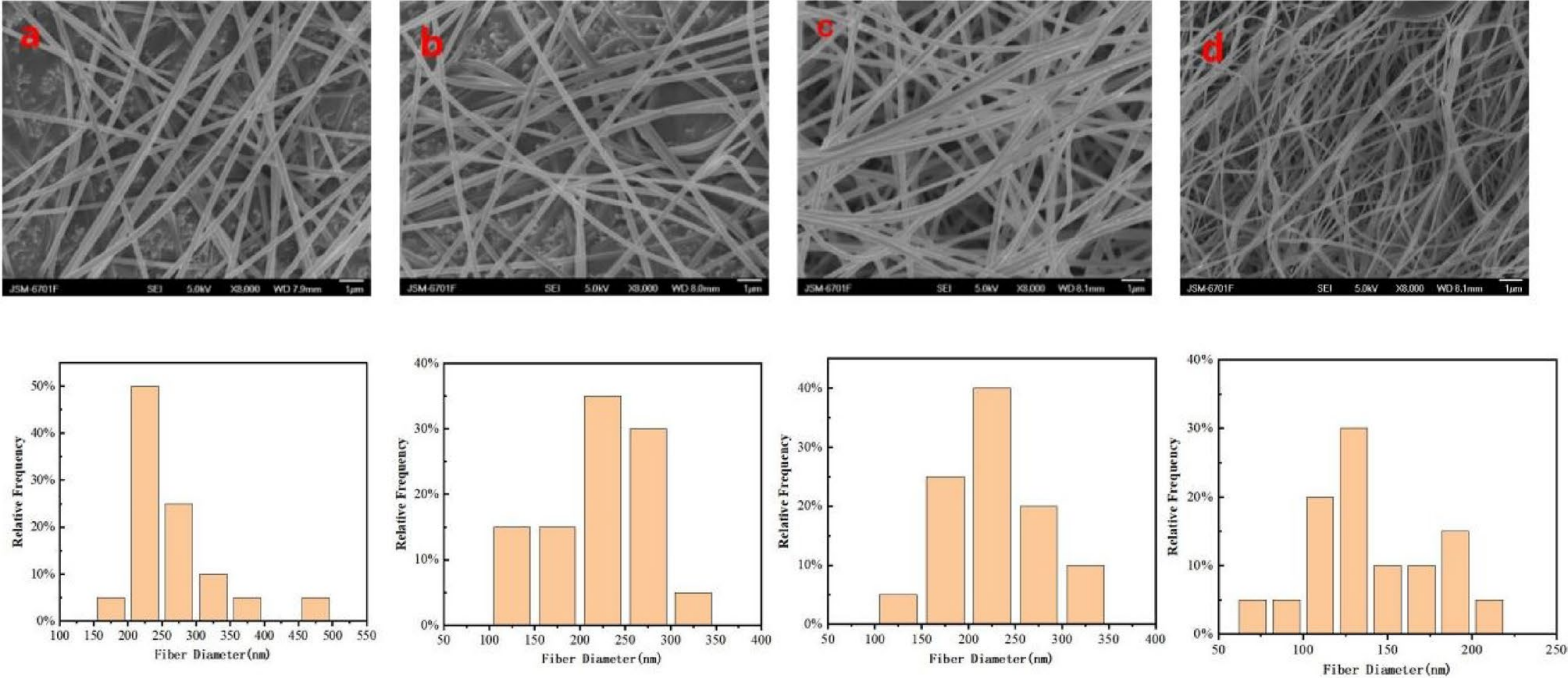
SEM images of electrospun CMKN fibers at different voltages and fiber diameters ((**a**):15 kV; (**b**):18 kV; (**c**):21 kV; (**d**):25 kV).

Figure 3 shows the SEM and fiber diameter distribution histogram of the nanofibers prepared by the spinning solution at 15, 18 kV and 25 kV. As shown in the figure, regular nanofibers can be spun within the voltage range of 15–25 kV. However, the fibers spun at 15 kV (Fig. 3a) and 18 kV (Fig. 3b) still had certain beads, and the spinning effect was unstable. In contrast, nanofibers spun at 21 kV (Fig. 3c) exhibited high uniformity and few disorderly fibers, and the diameter of the fibers was generally distributed between 200 and 250 nm. Meanwhile, the observation of the fibers spun at 25 kV (Fig. 3d) revealed that the fiber diameter distribution widened; although the fiber became fine for a short time, the uniformity decreased.

In electrospinning, the fiber diameter increases with the increase of voltage, but when the voltage exceeds a certain voltage, the fiber diameter will decrease. This phenomenon is caused by the increase of voltage and the distortion of liquid droplets. And the bending viscoelastic force is overcome by electricity. Taylor cone stretching leads to the increase of charge density on the jet surface, and the repulsive force caused by it leads to the jet splitting. As the diameter decreases further, the smaller jet splits. Therefore, the diameter of fiber increases as the voltage increases. However, when the voltage exceeds a certain range, the jet instability will decrease with the increase of voltage, leading to the decrease of the Taylor cone. Leading to a reduction in fiber diameter.

The electric field force increased with the voltage, causing the solution ejection speed to increase sharply. The spinning solution was sprayed on the receiver without good stretching in the electric field within a short time, making the overall effect of the fiber gap large, indicating that it is unsuitable for high voltage. Thus, 21 kV is suitable for the electrostatic spinning of CNKC. According to the above analysis, 21 kV voltage was selected for the follow-up study.

#### Effect of liquid pushing speed on electrospinning

**Figure 4:**
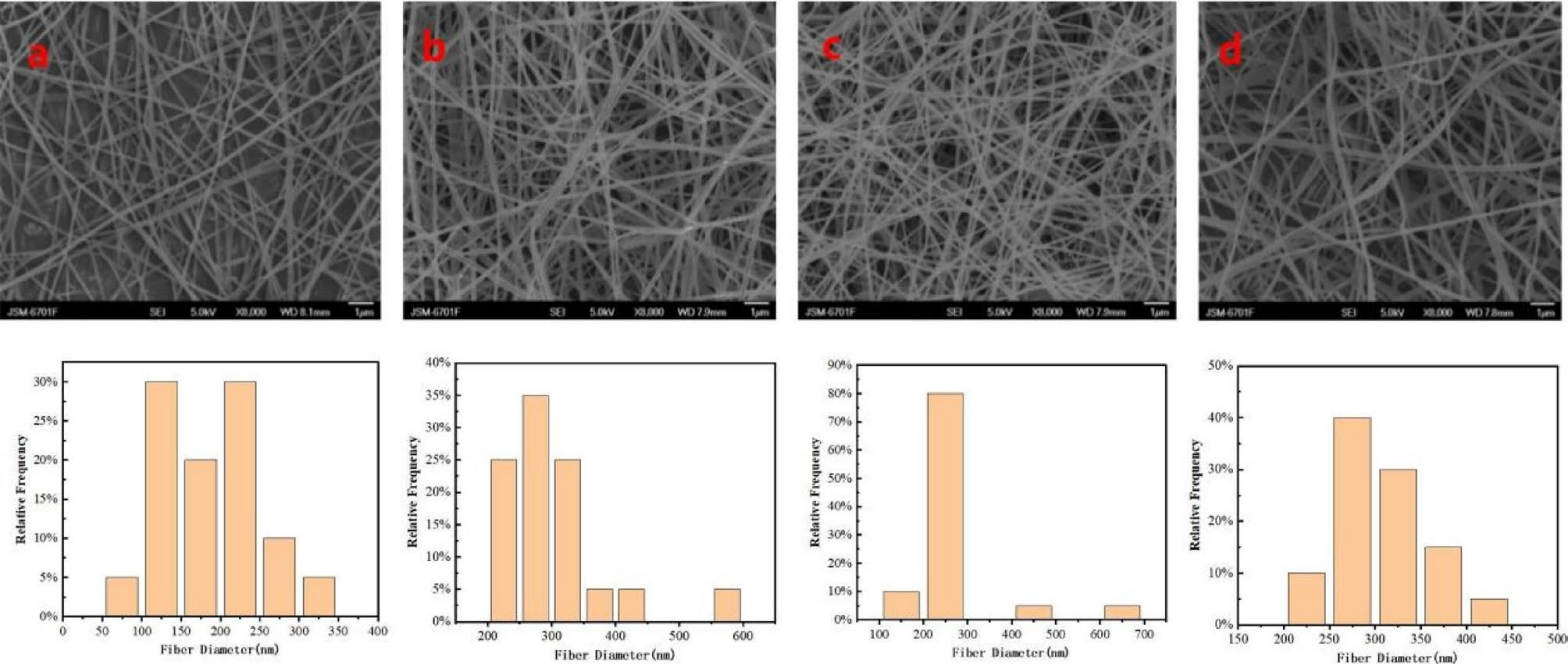
SEM images of electrospinning CMKN fibers at different pushing speed and fiber diameter distribution ((**a**):0.2 ml/h; (**b**):0.5 ml/h; (**c**):0.8 ml/h; (**d**):1 ml/h).

Meanwhile, selecting a reasonable pushing speed is also the key factor affecting the morphology of the electrostatic spinning fiber. In electrostatic spinning, the fiber with a low flow rate is fully polarized, and the morphology of the fiber is good^35^. According to the above analysis, we selected 15% cellulose spinning solution, 21 kV spinning voltage, and 20 cm receiving distance as the fixed parameters for exploring the influence of different pushing speeds on the electrostatic spinning fiber morphology. As shown in Fig. 4, the pushing velocity is 0.2, 0.5, 0.8 and 1 ml/h, respectively. The overall morphology and diameter distribution of the fiber are the same, and the main diameter of the fiber is basically distributed between 250 and 300 nm.

Generally, the polymer solution has sufficient polarization time, so it is recommended to use a lower flow rate. When the flow rate is high, because the drying time before reaching the collector is short and the tension is small, the bead fibers with larger diameter will be formed, rather than smooth fibers with smaller diameter.

From 0.2 to 1 mL/h, the fiber output increased gradually with the increase of the liquid pushing speed. The fiber diameter distribution diagram indicates that when the fiber pushing speed increased, part of the fiber diameter gradually increased, and the overall fiber uniformity decreased. Moreover, increasing the fiber pushing speed will have a great influence on the fiber morphology, forming filaments in the jet and even beads on the fiber of the receiver and decreasing the stability of electrostatic spinning^36^. Through experimental research and analysis, we selected the liquid pushing speed of 0.5 ml/h as the best parameter. At this speed, the fiber fineness is relatively neat, that is, mainly between 250 and 300 nm, and the surface is smooth.

#### Effect of receiving distance on electrospinning

**Figure 5:**
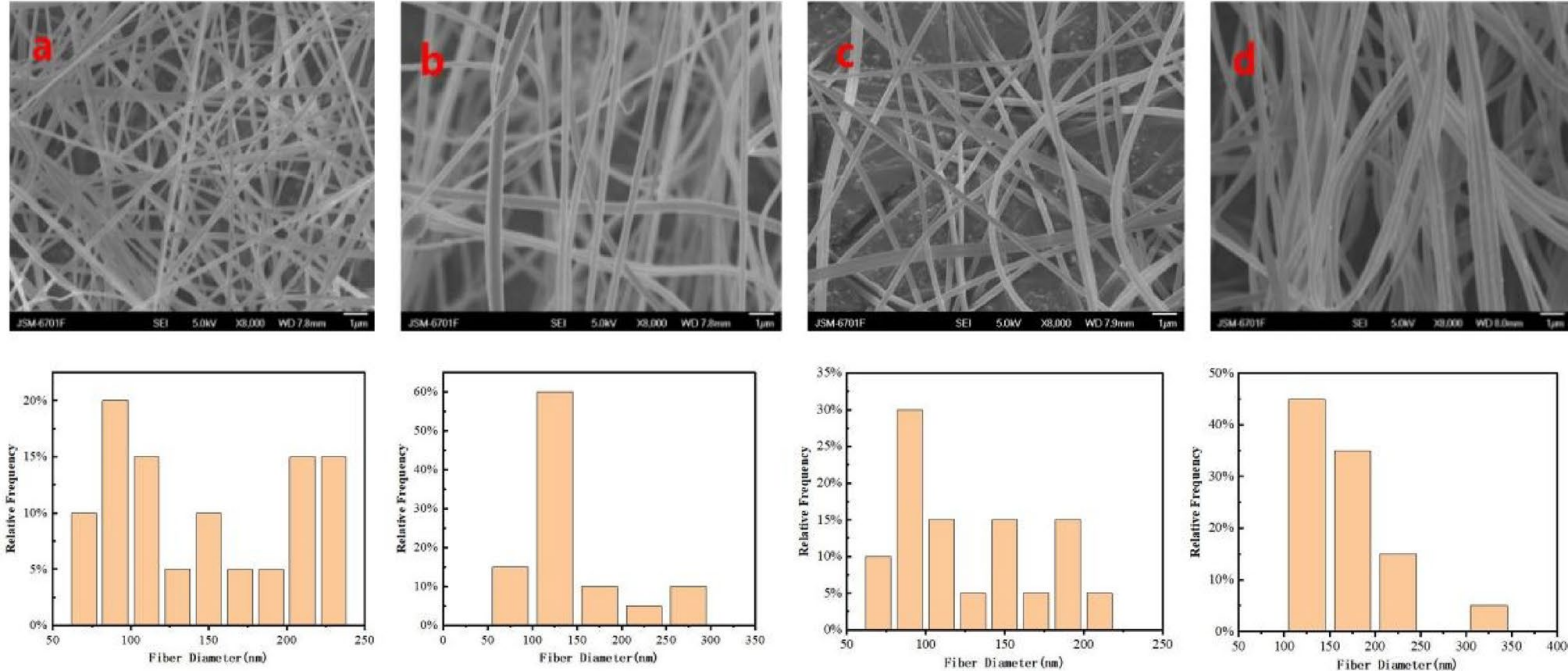
SEM images of electrospun CMKN fibers at different collection distances and fiber diameter distribution ((**a**):10 cm; (**b**):15 cm; (**c**):20 cm; (**d**):25 cm).

Figure 5 shows the SEM images and fiber distribution histograms of the spinning solution with 15% cellulose ratio at 21 kV, 0.5 mL/h pushing speed, and different receiving distances. In this experiment, 10 cm (Fig. 5a), 15 cm (Fig. 5b), 20 cm (Fig. 5c) and 25 cm (Fig. 5d) were selected for reference.

The figure shows that when the receiving distance of the fiber was 10 and 15 cm, the fiber morphology showed no obvious problems, but the fiber diameter distribution was wide, the thickness gap was obvious, and the fiber was not neat. At the receiving distance of 20 cm, the overall uniformity of the fiber improved significantly, and the fiber diameter was mainly concentrated between 60 and 100 nm. When the fiber was lit 25 cm, the fiber began to become coarser and the overall uniformity decreased. As the receiving distance increased, the voltage remained constant and the electric field intensity decreased^37^. The jet velocity decreased, and the fiber was not fully stretched and did not thicken. The range is extremely small and will cause inadequate fiber stretching and incomplete solvent volatilization, resulting in fiber adhesion and pile knot.

The distance from the tip of the receiver also affects the diameter and morphology of the fiber. In short, if the distance is too short, the fiber does not have enough time to cure before reaching the collector, while if the distance is too long, the bead fiber can be obtained. An important physical property of electorspun fiber is solvent drying. Therefore, the fiber surface morphology at the 20 cm receiving distance (Fig. 5c) is better than that at other distances. Furthermore, the spinning effect is better with higher uniformity and good fiber orientation at the 20 cm receiving distance than in the other distances. Therefore, 20 cm is the best receiving distance.

### XRD analysis

The crystallinity of fiber can be determined by XRD analysis^38^. Fiber crystallinity is commonly used to measure the mechanical properties of fiber, that is, the higher the crystallinity of the fiber is, the better the relative mechanical properties are; but an extremely high crystallinity will lead to fiber rigidity, that is, elasticity reduction^39^. In the diffraction pattern, the crystalline and amorphous regions of the fiber can be inferred from the XRD structure^40^.

**Figure 6:**
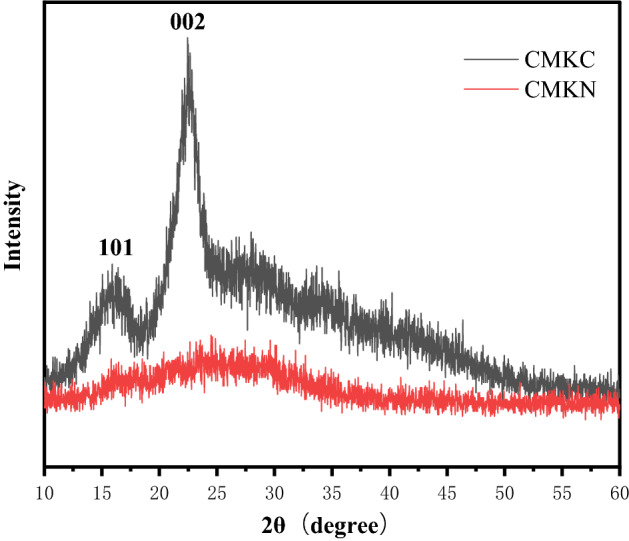
X-ray diffractograms of CMKC and CMKN.

Figure 6 shows the XRD patterns of CMKC and CMKN and the diffraction patterns of fiber changes under XRD. The initial CMKC was transformed into CMKN, and the crystal ratio changed. Figure 6 shows that the main crystal peak occurred when CMKC 2θ = 22–23°, which corresponds to cellulose crystal type **I**^41^. In addition, the spectra of CMKN and CMKC have similar variation trends and diffraction peak intensities. However, the crystallization index of CMKN, which becomes cellulose nanofibers after treatment, is much lower than that of CMKC before treatment. During electrospinning, the crystal structure of natural cellulose was obviously disturbed, decreasing the crystallinity after the deposition of nanofibers^42^. When the crystallinity of fiber is affected by mechanical or chemical factors, the hydrogen bonds between cellulose segments will be destroyed, and the crystallinity of CMKN will be much lower than that of CMKC^43^.

The main diffraction peaks of 2θ = 15.8° and 22.9° appear in the diffraction pattern of CMKC samples. This is the characteristic of the lattice of type I cellulose 101 and 002, corresponding to the primary crystal structure of cellulose. In the process of electrospinning, the crystal structure of the natural cellulose is disordered, and the crystallinity of the nanofibers is obviously reduced during deposition. The amorphous phase in nanofibers occurs because most of the hydrogen bonds break the type I crystal structure as the cellulose dissolves. In the CMKN diffraction pattern, two peaks (2θ = 16.5° and 23.5°) are also observed, which may preserve part of the natural cellulose crystal structure.

### FT-RT analysis

**Figure 7:**
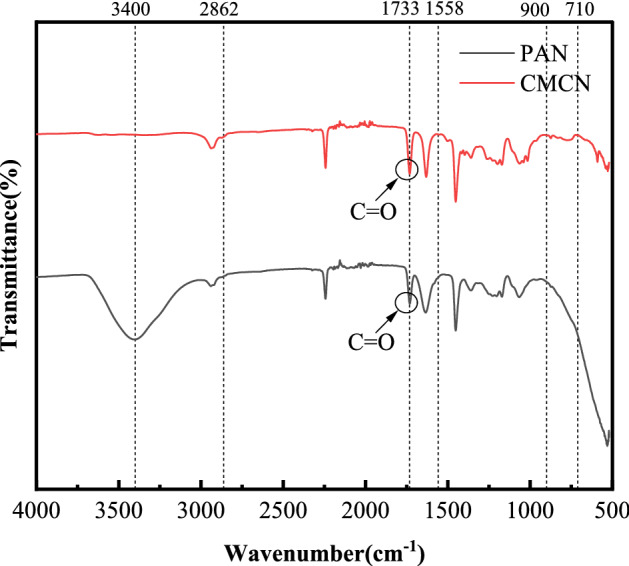
FTIR spectra of PAN and CMKN.

Infrared spectrometer is used for analyzing the molecular structure and the chemical composition on the basis of the absorption characteristics of different wavelengths of infrared radiation^44^. In Fig. 7, we mainly compare and analyze the changes of the infrared spectrum of CMKN after PAN and electrostatic spinning.

The figure shows that certain changes occurred in the structure of cellulose to nano fiber. In the CMKN spectrum, a strength band formed by O–H bond stretching was observed at 3400 cm^−1^, and a medium strength band formed by C–H bond stretching was observed at 2862 cm^−1^^45^. The changes at 1733 and 1558 cm^−1^ were due to the disappearance of the glycofurfural ester and acetyl hemicellulose or carboxyl ester bond lignin in the fiber^46^. This result indicates that our nanofibers effectively removed some non-cellulose components and increased the cellulose components compared with the original product. The absorbance of 1330–970 cm^−1^ in the CMKN region is due to the stretching of the C–O bond^47^, and the 900 cm^−1^ band is characteristic of the β-glycoside bond between sugar units^38^. The peaks of 1421, 1364, 1323, 1142, 1050, 1029, and 900 cm^−1^ are significantly correlated with the cellulose peaks^48^. A small band was found at 712 cm^−1^, which is the type I form of cellulose^49^, which is currently the dominant cellulose form of PAN. The changing structure of the infrared spectrum shows that after chemical or physical treatment, some non-cellulose structures were effectively removed, making the composition of cellulose prominent, and the form of cellulose is cellulose I.

### Mechanical and physical test

**Figure 8:**
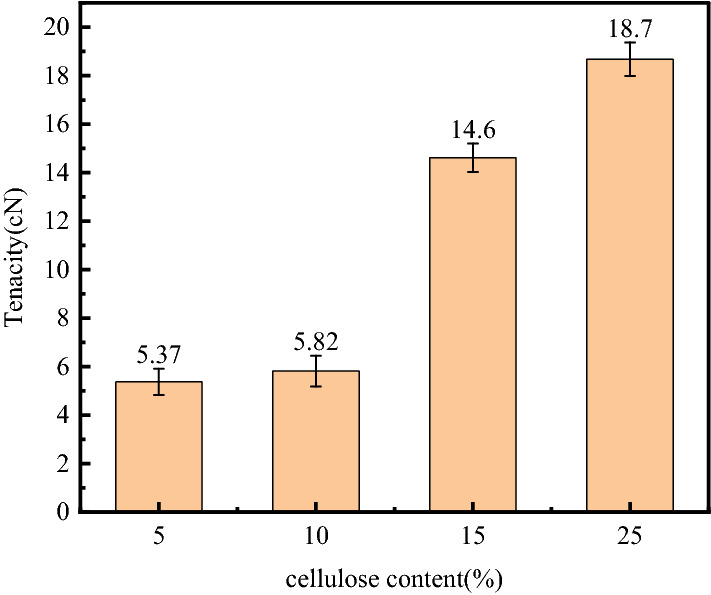
Tensile strength of a spinning fluid with different cellulose content.

Given the small size of electrospun nanofibers, no specific method is suitable for measuring them. Therefore, a single-fiber strength tensimeter is widely used to measure the mechanical properties of nanofiber membranes^27^. The tensile strength of materials can be measured by their physical quantities, such as the maximum tensile strength, the breaking force, the elongation at break, and the elastic modulus^50^. Figure 8 shows that this experiment compares the effects of different proportions of the cellulose/PAN spinning solution on the mechanical properties of CMKN under 21 kV, the pushing speed of 0.5 mL/h, and the receiving distance of 20 cm.

The figure shows that the strength of the spinning solutions with different cellulose ratios also varies. The mechanical properties are greatly improved with the increase of the cellulose ratio in the spinning solution. The strength of the CMKN spun from 5 and 10% spinning solutions did not improve significantly, but the mechanical properties began to improve greatly when it was added to 15%. This phenomenon is due to the fact that very small cellulose content usually has almost no effect on spinning solution. Insufficient ultrasonic time leads to uneven mixing of spinning solution. Adding cellulose can improve the mechanical properties of the film, but too much cellulose will lead to poor spinning effect and many defects in the fiber appearance, which will lead to the inability to spin normally.

### Dye removal rate analysis

#### Single factor experimental analysis of dye removal rate

**Figure 9:**
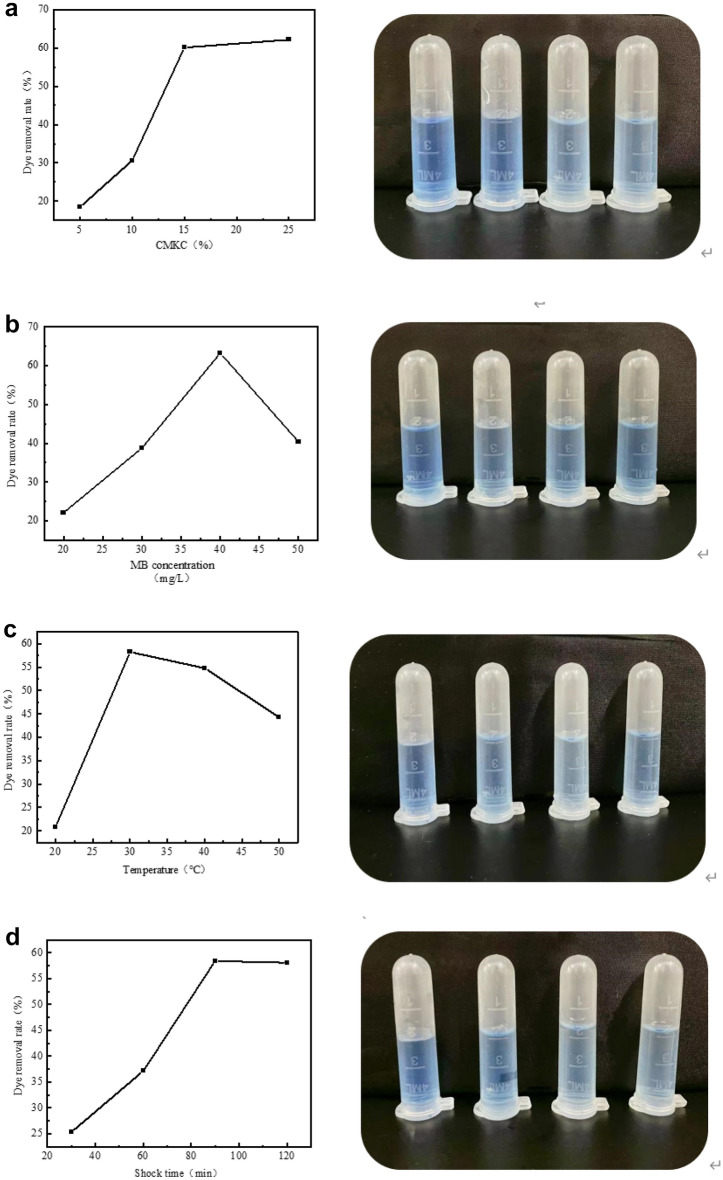
Effect of different factors on dye removal rate ((**a**): CMKC ratio; (**b**): MB concentration; (**c**): Temperature; (**d**): shock time).

As shown in Fig. 9a, Under the conditions of 30 °C, methylene blue (MB) concentration of 40 mg/L, adding 2.5 mol/L H_2_O_2_, shaking for 90 min, 0.5 mg carex meyeriana kunth cellulose nanomembrane was selected, and the cellulose content in the membrane was divided into 5%, 10%, 15% and 25% for the experiment. As can be seen from Table 1, the dye removal rate will increase with the increase of cellulose ratio, but the performance of 25% nanofilm is not good enough. Therefore, the nanomembranes with a cellulose ratio of 15% were selected for discussion.

As shown in Fig. 9b, cut 0.5 mg nanomembrane with 15% cellulose content, add 2.5 mol/L H_2_O_2_ in methylene blue (MB) concentration of 40 mg/L dye solution, shake for 90 min, the above conditions are fixed. The temperature is set to 20 °C, 30 °C, 40 °C, 50 °C, etc. The dye removal rate can be seen from the table. When the temperature is below 30℃, the dye removal rate can only reach about 20%, and when it reaches 30 °C, the dye removal rate is significantly increased. The dye removal rate gradually decreased after 30 °C. When the temperature is changed, the ions will move faster with the increase of temperature, and the dye will quickly find the attachment point of the film. However, when the attachment point is basically coated by the dye, the removal rate will be stable, and the adsorption effect will not increase significantly. Rising temperature will also decompose H_2_O_2_, leading to a decrease in removal rate. Therefore, the next 30 °C will be selected for the experiment.

As shown in Fig. 9c, at 30 °C, 0.5 mg nanofilm with 15% cellulose added, 2.5 mol/L H_2_O_2_ added, methylene blue (MB) concentration selected 40 mg/L dye solution for the test, the above conditions are fixed. The shock time is set to 30 min, 60 min, 90 min and 120 min respectively. As can be seen from the table, the dye removal rate gradually increased with the increase of the oscillation time, but after 90 min, the removal rate did not increase, but decreased to a certain extent. In the process of adsorption, the dye fully binds to the binding point on the membrane surface with the increase of time, but when the binding point is completely occupied, the adsorption amount of dye will not rise and a certain degree of dissociation will occur. After that, no matter how the adsorption time increases, the removal rate will not be affected, and the adsorption and dissociation of the dye will reach a dynamic equilibrium.

As shown in Fig. 9d, at 30 °C, 0.5 mg nanofilm containing 15% cellulose was used, 2.5 mol/L H2O2 was added, and the shaking time was 90 min. The above conditions were fixed. Dye solution methylene blue (MB) concentration of 20 mg/L, 30 mg/L, 40 mg/L and 50 mg/L were selected for the test. As can be seen from the table, between the concentration of dye solution and the removal rate, the removal rate will increase with the increase of concentration, but the removal rate will decrease when the concentration exceeds a certain range. Since the dye concentration is low and the attachment points are many, increasing the concentration will increase the dye, so as to effectively adhere to the membrane surface. However, if the concentration is too high, the dye will increase, and the attachment point is limited, and the solution is too concentrated, the dye will not spread well, leading to the poor adsorption effect of the film, and the removal rate will decrease.

#### Response surface analysis

According to the single factor test, the optimal experimental range of significant factors was selected, and the total dye removal rate was taken as the optimization index CMKN adsorption MB staining solution process conditions for response surface analysis, so as to optimize the process again. The experimental results, regression equation diagram and response surface analysis of variance are shown in Table 2 and Table 3.

**Table 2:** Box-Behnken Design Test Results.

No	Temperature (°C)	Shock time (min)	MB concentration (mg/L)	Dye removal rate (%)
1	30	80	50	47.81
2	30	90	40	63.24
3	25	80	40	45.08
4	25	90	30	44.79
5	30	100	30	58.04
6	30	90	40	61.16
7	30	90	40	62.72
8	30	90	40	62.87
9	30	90	40	63.21
10	30	80	30	57.82
11	35	100	40	55.52
12	35	90	50	52.58
13	30	100	50	53.94
14	25	100	40	40.88
15	35	90	30	51.35
16	25	90	50	37.08
17	35	80	40	50.67

**Table 3:** Analysis-of-variance results.

Source	Sum of squares	Degree of freedom	Mean square	F	P
Model	1069.38	9	118.82	54.65	< 0.00001
Residual	15.22	7	2.17		
Lack of fit	12.28	3	4.09	5.58	0.0651
Pure error	2.93	4	0.7337		
Cor total	1084.60	16			

**Figure 10:**
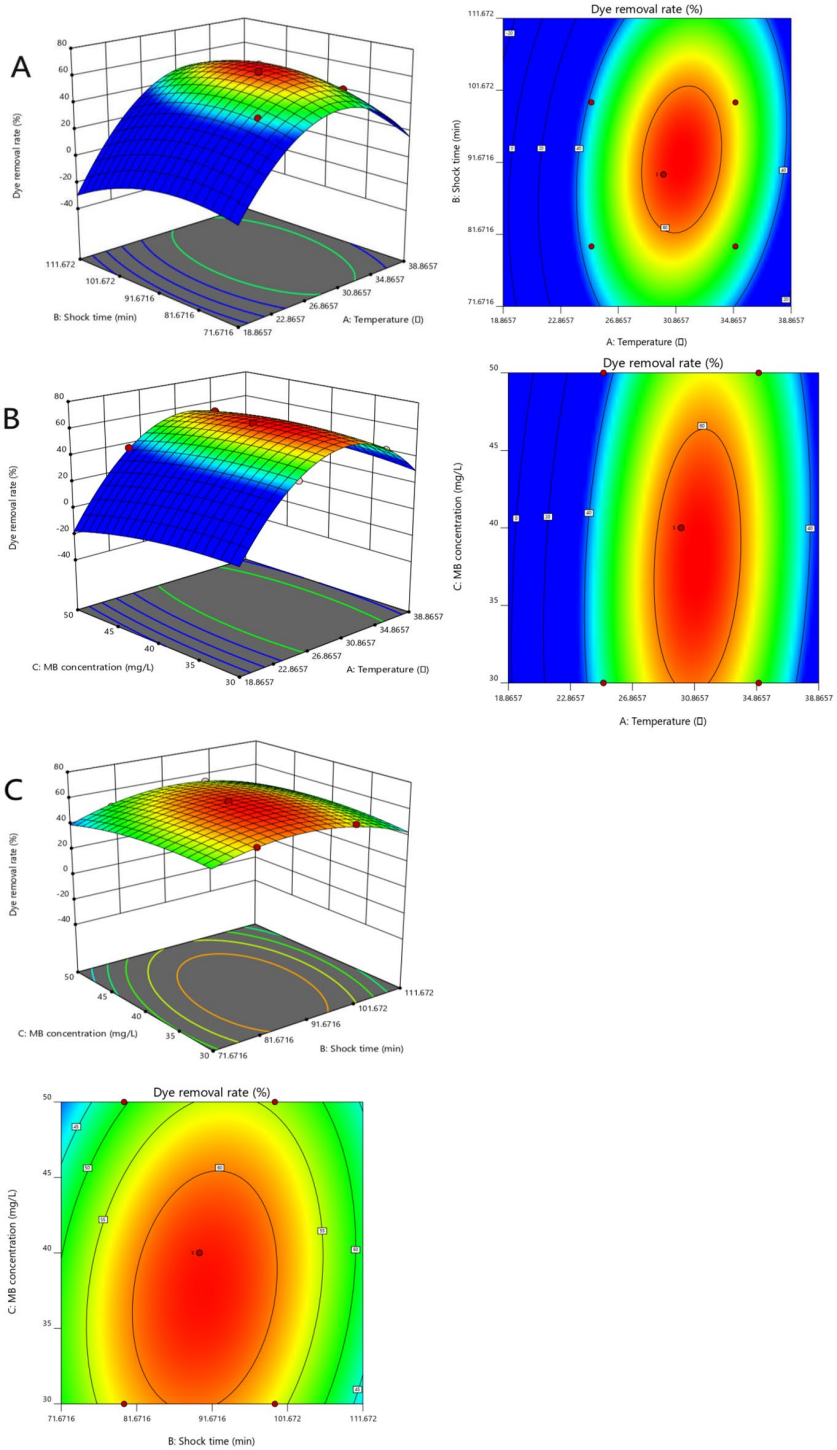
Response surface plot and contour plot of the influence of Various factors on removal rate ((**a**): Temperature and Shock time; (**b**): MB concentration and Temperature; (**c**): MB concentration and Shock time).

Figure 10 shows the interaction of three factors: shock time, MB concentration and temperature. The significant level of influencing factors can be seen from the surface conditions and contour plots of each figure. The steep surface indicates that the influencing factors are significant, and the shape of the contour line can reflect the strength of the interaction effect. The oval indicates that the interaction between the two factors is significant, while the circle indicates that the interaction effect is not obvious^51^. The surface in Fig. 10 is steep and the contour lines approach the ellipse, indicating that the interaction effect is significant.

Combined with the above analysis, the software was used for further analysis, and the optimal experimental conditions were as follows: shock time 90 min, MB concentration 40 mg/L, temperature 30 °C, removal rate of 63.24%. In order to validate the result of the prediction, using the optimal conditions for experiments, the removal rate of CMKN, comparing the regression equation of predicted value, its deviation was 0.6%, the fitting of the correlation between experimental value and predicted value is good, prove that the conditions of the model analysis and prediction is more accurate and reliable, has certain practical value.

Multiple regression analysis was conducted on the experimental results of CMKN adsorbing MB dye. According to the variance analysis results of each item in the regression equation (Table 2), F value of Model was 54.65, P value was < 0.0001, indicating that Model was extremely significant. A (temperature), B (shock time) and C (MB concentration) were significant factors, and the order of influence of each factor on MB removal rate was as follows: A > B > C. Among them, the interaction between AB and AC was significant, and the influence of the other terms was not significant enough, indicating that the relationship between each factor and the response value could not be explained by a simple linear relationship, but a nonlinear relationship. The correlation coefficient R^2^ of the model is 0.9860, 98.60% of the variation in response value was due to the three selected variables. And F value of the misfitting term is 5.58, P value is 0.0651, indicating that the model has a high degree of fitting to the actual situation, and the regression equation can be used the removal rate of MB solution under different experimental conditions was predicted and the optimal experimental conditions were obtained. According to the results of regression analysis (Table.2) Make the corresponding surface diagram.

## Conclusions

In this study, the cellulose fibers of carex meyeriana kunth were prepared by electrospinning with cellulose of carex meyeriana kunth as raw material. The influence of electrospinning parameters, such as CMKC concentration, spinning voltage, receiving distance and liquid pushing speed, on the formation of CMKN was explored through univariate experiments. The diameter of electrospun fiber membrane was measured, and the influence of electrospinning parameters on fiber diameter was explored. It is found that the best CMKN can be obtained under the experimental conditions of voltage 21 kV, receiving distance 20 cm and liquid pushing speed 0.5 ml/h.

CMKN was tested and characterized by scanning electron microscope, XRD, infrared spectrum and tensile strength test of single fiber. The results show that the CMKN spun under the optimum process parameters has smooth fiber surface, high overall orientation and strong uniformity. Compared with the original PAN electrospinning membrane, the cellulose content in CMKN is obviously increased, and the cellulose of CMKN prepared is type I. The crystallinity of cellulose of carmeyeiana Kunth decreased after being converted into nanofibers. The increase of cellulose content in CMKN can effectively improve the mechanical strength of nanofiber membrane.

For the experiment of CMKN on dye removal, we discussed the effects of single factors such as CMKC addition, temperature, oscillation time and MB initial concentration on dye removal rate. Using BBD design, taking temperature, oscillation time and initial MB concentration as influencing factors, and MB removal rate as response value, the response surface quadratic equation model is very significant. Response surface methodology is helpful to understand the interaction of CMKC dosage, temperature, oscillation time and MB initial concentration. Langmuir adsorption isotherm is the best fitting model for MB removal by CMKN. The pseudo-second-order model can better explain the dynamic model. According to the experiment, the best dye removal condition is 30℃, MB solution concentration 40 mg/L, impact time 90 min, and the maximum MB removal rate of CMKN with 15%CMKC is 63.24%.

The results show that the nanofiber prepared by combining carex meyeriana kunth cellulose with the existing electrospinning method has good performance, strong dye removal ability and good application prospect. This research has opened up a new development direction for the application of natural carex meyeriana kunth resources, improved the commercial potential of carex meyeriana kunth, and made a new exploratory attempt to alleviate the fossil energy consumption and the pressure of the earth environment.

## Data Availability

The datasets used and/or analysed during the current study available from the corresponding author on reasonable request.
